# Advancements in dementia research, diagnostics, and care in Latin America: Highlights from the 2023 Alzheimer's Association International conference satellite symposium in Mexico City

**DOI:** 10.1002/alz.13850

**Published:** 2024-05-27

**Authors:** Ana Luisa Sosa, Sonia MD Brucki, Lucia Crivelli, Francisco Javier Lopera, Daisy M Acosta, Juliana Acosta‐Uribe, Diego Aguilar, Sara G Aguilar‐Navarro, Ricardo F Allegri, Paulo HF Bertolucci, Ismael L Calandri, Maria C Carrillo, Patricio Alexis Chrem Mendez, Mario Cornejo‐Olivas, Nilton Custodio, Andrés Damian, Leonardo Cruz de Souza, Claudia Duran‐Aniotz, Adolfo M García, Carmen García‐Peña, Mitzi M Gonzales, Lea T Grinberg, Agustin M Ibanez, Maryenela Zaida Illanes‐Manrique, Clifford R Jack, Jorge Mario Leon‐Salas, Jorge J Llibre‐Guerra, José Luna‐Muñoz, Diana Matallana, Bruce L Miller, Lorina Naci, Mario A Parra, Margaret Pericak‐Vance, Stefanie D Piña‐Escudero, Elisa de Paula França Resende, John M Ringman, Gustavo Sevlever, Andrea Slachevsky, Claudia Kimie Suemoto, Victor Valcour, Andres Villegas‐Lanau, Mônica S Yassuda, Simin Mahinrad, Claire Sexton

**Affiliations:** ^1^ Laboratorio de Demencias del Instituto Nacional de Neurología y Neurocirugía Manuel Velasco Suárez Mexico City CDMX México; ^2^ Department of Neurology Cognitive and Behavioral Group University of Sao Paulo São Paulo São Paulo Brazil; ^3^ Department of Cognitive Neurology Fleni Buenos Aires Argentina; ^4^ Grupo de Neurociencias (GNA) Facultad de Medicina Universidad de Antioquia Medellín Colombia; ^5^ Universidad Nacional Pedro Henriquez Urena (UNPHU) Santo Domingo Dominican Republic; ^6^ Neuroscience Research Institute and Department of Molecular Cellular and Developmental Biology University of California Santa Barbara Santa Barbara California USA; ^7^ Alzheimer's Disease International London UK; ^8^ Department of Geriatrics Instituto Nacional de Ciencias Médicas y Nutrición Salvador Zubiran México CDMX Mexico; ^9^ Instituto de Neurociencias Fleni Buenos Aires Argentina; ^10^ Department of Neurosciences Universidad de la Costa CUC Barranquilla Colombia; ^11^ Neurology & Neurosurgery Department Escola Paulista de Medicina/UNIFESP São Paulo São Paulo Brazil; ^12^ Alzheimer's Association Chicago Illinois USA; ^13^ Memory Center Fleni Buenos Aires Argentina; ^14^ Neurogenetics Working Group Universidad Científica del Sur Lima Peru; ^15^ Neurogenetics Research Center Instituto Nacional de Ciencias Neurologicas Lima Peru; ^16^ Unidad de Diagnóstico de Deterioro Cognitivo y Prevención de Demencia Instituto Peruano de Neurociencias Lima Peru; ^17^ Escuela Profesional de Medicina Humana Universidad Privada San Juan Bautista Lima Peru; ^18^ Departamento de Montevideo Centro Uruguayo de Imagenología Molecular (CUDIM) and Unidad Académica de Medicina Nuclear e Imagenología Molecular Hospital de Clínicas Universidad de la República (UdelaR) Montevideo Montevideo Uruguay; ^19^ Programa de Pós‐Graduação em Neurociências Universidade Federal de Minas Gerais (UFMG) Belo Horizonte MG Brazil; ^20^ Departamento de Clínica Médica da Faculdade de Medicina da UFMG Belo Horizonte MG Brazil; ^21^ Latin American Brain Health Institute (BrainLat) Universidad Adolfo Ibañez Santiago Chile; ^22^ Center for Social and Cognitive Neuroscience (CSCN) School of Psychology Universidad Adolfo Ibañez Santiago Chile; ^23^ Global Brain Health Institute (GBHI) University of California San Francisco (UCSF) San Francisco California USA; ^24^ Cognitive Neuroscience Center Universidad de San Andrés Victoria Provincia de Buenos Aires Argentina; ^25^ Departamento de Lingüística y Literatura Facultad de Humanidades Universidad de Santiago de Chile Santiago Chile; ^26^ Instituto Nacional de Geriatría México CDMX Mexico; ^27^ Department of Neurology Cedars Sinai Medical Center Los Angeles California USA; ^28^ Glenn Biggs Institute UT Health San Antonio San Antonio Texas USA; ^29^ Department of Neurology and Pathology University of California San Francisco (UCSF) San Francisco California USA; ^30^ Department of Pathology University of Sao Paulo São Paulo São Paulo Brazil; ^31^ Global Brain Health Institute (GBHI) University of Trinity Dublin Lloyd Building Trinity College Dublin Dublin Ireland; ^32^ Cognitive Neuroscience Center (CNC) Universidad de San Andrés, and CONICET Victoria Argentina; ^33^ Trinity College Dublin (TCD) College Green Dublin Ireland; ^34^ Mayo Clinic Rochester Minnesota USA; ^35^ Departamento de Investigación Clínica Life Science Research Institute Hospital Clinica Biblica San José Costa Rica; ^36^ Dominantly Inherited Alzheimer's Network Trials Unit Washington University School of Medicine St. Louis Missouri USA; ^37^ Department of Neurology Washington University School of Medicine in St.Louis St. Louis Missouri USA; ^38^ Banco Nacional de Cerebros‐UNPHU Universidad Nacional Pedro Henríquez Ureña Santo Domingo Dominican Republic; ^39^ National Dementia BioBank Dirección de Investigación Innovación y Posgrado Universidad Politécnica de Pachuca Zempoala México; ^40^ Federación Mexicana de Alzheimer (FEDMA) México CDMX Mexico; ^41^ Pontificia Universidad Javeriana Medical School Aging Institute Hospital Universitario San Ignacio Bogotá Colombia; ^42^ Memory and Cognition Center, Intellectus Hospital Universitario San Ignacio Bogotá Colombia; ^43^ Mental Health Department Hospital Universitario Fundación Santa Fe Bogotá Colombia; ^44^ Department of Neurology Memory and Aging Center University of California San Francisco (UCSF) San Francisco California USA; ^45^ Trinity College Institute of Neuroscience Trinity College Dublin College Green Dublin Ireland; ^46^ Department of Psychological Sciences and Health University of Strathclyde Glasgow UK; ^47^ John P Hussman Institute for Human Genomics Miller School of Medicine University of Miami Coral Gables Florida USA; ^48^ Dr. John T Macdonald Foundation Department of Human Genetics University of Miami Miller School of Medicine Coral Gables Florida USA; ^49^ Universidade Federal de Minas Gerais Faculdade de Medicina Ciências Médicas de Minas Gerais Hospital das Clínicas ‐ EBSERH‐UFMG Belo Horizonte Brazil; ^50^ Department of Neurology Alzheimer's Disease Research Center Keck School of Medicine University of Southern California Los Angeles California USA; ^51^ Department of Neuropathology Fleni Buenos Aires Argentina; ^52^ Geroscience Center for Brain Health and Metabolism (GERO) Santiago Chile; ^53^ Memory and Neuropsychiatric Center (CMYN) Neurology Department Hospital del Salvador & Faculty of Medicine University of Chile Santiago de Chile Chile; ^54^ Neuropsychology and Clinical Neuroscience Laboratory (LANNEC) Physiopathology Program – Institute of Biomedical Sciences (ICBM) Neuroscience and East Neuroscience Departments Faculty of Medicine University of Chile Santiago de Chile Chile; ^55^ Servicio de Neurología Departamento de Medicina Clínica Alemana‐Universidad del Desarrollo Las Condes Chile; ^56^ Division of Geriatrics University of Sao Paulo Medical School São Paulo São Paulo Brazil; ^57^ Department of Neurology University of California San Francisco (UCSF) San Francisco California USA; ^58^ Gerontology, School of Arts Sciences and Humanities University of Sao Paulo São Paulo São Paulo Brazil

**Keywords:** Alzheimer's disease, biomarkers, dementia, genetics, Hispanics, Latin America

## Abstract

**INTRODUCTION:**

While Latin America (LatAm) is facing an increasing burden of dementia due to the rapid aging of the population, it remains underrepresented in dementia research, diagnostics, and care.

**METHODS:**

In 2023, the Alzheimer's Association hosted its eighth satellite symposium in Mexico, highlighting emerging dementia research, priorities, and challenges within LatAm.

**RESULTS:**

Significant initiatives in the region, including intracountry support, showcased their efforts in fostering national and international collaborations; genetic studies unveiled the unique genetic admixture in LatAm; researchers conducting emerging clinical trials discussed ongoing culturally specific interventions; and the urgent need to harmonize practices and studies, improve diagnosis and care, and use affordable biomarkers in the region was highlighted.

**DISCUSSION:**

The myriad of topics discussed at the 2023 AAIC satellite symposium highlighted the growing research efforts in LatAm, providing valuable insights into dementia biology, genetics, epidemiology, treatment, and care.

## 1 INTRODUCTION

The prevalence of Alzheimer's disease and related dementias (ADRD) is growing rapidly across the globe, posing significant health and economic challenges. A coordinated global response, coupled with prioritizing research efforts, is imperative to effectively address the global burden of ADRD.^1^, ^2^ Since 2015, the Alzheimer's Association International Conference (AAIC) has hosted AAIC satellite symposiums in various world regions to highlight regional research advancements in ADRD and foster international collaborations aimed at identifying research gaps and priorities for diagnosis, prevention, and treatment. In 2023, the eighth AAIC Satellite Symposium was hosted in partnership with the Global Brain Health Institute (GBHI) in Mexico City, Mexico, to explore emerging approaches, priorities, and challenges in ADRD research within Latin America (LatAm).

Latin American countries are encumbered by increased ADRD prevalence and incidence due to a rapid demographic shift with accelerated growth of the aging population.^2^, ^3^ Such trends place profound healthcare, social, and economic burdens on patients, caregivers, public health, and social systems.^4^, ^5^ A growing body of evidence also highlights the underrepresentation of vulnerable populations in LatAm in dementia research and healthcare settings.^6^, ^7^ In recognition of the challenges and scientific opportunities in the region, the research landscape in LatAm has achieved a four‐fold increase in funding from the Alzheimer's Association since the first AAIC satellite symposium in Mexico City in 2015. Indeed, in 2023, the Alzheimer's Association funding supported 41 active research grants totaling over $4 million. This funding includes the Multi–Partner Consortium to Expand Dementia Research in LatAm (ReDLat Consortium, supported by the National Institutes of Health‐National Institute on Aging [NIH‐NIA], the Alzheimer's Association, the Rainwater Charitable Foundation, The Bluefield Project, Takeda, Alector, and GBHI), the Latin America Initiative for Lifestyle Intervention to Prevent Cognitive Decline (LatAm‐FINGERS), which is taking place simultaneously in 12 LatAm countries, and the Dominantly Inherited Alzheimer's Disease Network in Latin America (DIAN‐LatAm) operating in four countries, among others.

In this review, we provide a summary of the topics discussed at the 2023 AAIC satellite symposium in Mexico City. The topics included epidemiological research on dementia, the development and implementation of national dementia plans (NDPs), and molecular, genetic, and biomarker advancements within LatAm. Other topics included the study of social determinants, Latin American initiatives, harmonizing and sharing data to develop collaborative research, strategies to reach diverse populations, progress in pharmacological and non‐pharmacological interventions, and clinical trial experience in LatAm. The contribution of Biobanks to dementia science and their organization into networks in LatAm and the Caribbean were also discussed.

## 2 EPIDEMIOLOGY

The global population is aging due to an accelerated increase in life expectancy and a decline in fertility rates. This demographic transition is occurring faster in LatAm than in high‐income countries (HICs). Comparison of the population pyramids in LatAm between 1950 and 2022 reveals a continuous population growth in all age groups over a period spanning 70 years.^8^ Over this period, the life expectancy in the region has increased from 48.6 years in 1950 to 75.2 years in 2019, and it is projected to be more similar to the life expectancy in North America by 2100. Consequently, LatAm is experiencing a rapid aging phase, with projections suggesting that by 2047, individuals aged 60 and over will outnumber those under 15 years of age. These demographic transitions have profound implications and present challenges, such as a heightened prevalence of multimorbidity in the region.^8^

Epidemiologic studies on the prevalence of dementia in LatAm have reported varying prevalence estimates in different regions, primarily due to methodological differences. However, the 10/66 Dementia Research Group (DRG) has shown more consistent results by using the same protocols, clinical assessments, and validated instruments for low‐ and middle‐income countries (LMICs). Their findings demonstrate that the prevalence of dementia across LatAm is more similar than previously reported, ranging from 6.7% to 12.6% in 2008.^2^, ^9^, ^10^ According to the Global Burden of Disease (GBD), the prevalence of dementia in LatAm in 2019 was the highest in Argentina, Chile, and Uruguay.^2^ A recent systematic review and meta‐analyses reported that the prevalence of all‐cause dementia in LatAm is 8% to 10%, with a higher prevalence for women, lower‐educated individuals, and rural residents.^11^ The GBD estimates also suggested a 205% increase in the number of people aged 60 and above in the region between 2010 and 2015, compared to a 102% increase in North America.^2^ Looking into the incidence of dementia in LatAm, the incidence rates range from 18.2 in Peru to 30.4 in Mexico per 1000 person‐years for people aged 65 years and older.^3^ In Brazil, the incidence varies from 11.2 to 26.1 per 1000 person‐years.^12^, ^13^, ^14^

The impact of dementia can be assessed from a number of indicators, such as mortality rates, disability‐adjusted life years (DALYs), and annual costs. There was an increase in ADRD mortality rates in LatAm between 1990 and 2019.^2^ Dementia ranked as the eighth and fourth leading cause of DALYs among men and women aged 70+ years, respectively, between 2000 and 2019.^2^ According to GBD, the annual cost of dementia per person in Central, Southern, and Tropical regions of LatAm increased by up to 87% between 2010 and 2015, while the Andean region experienced a decline in dementia annual costs.^2^ Furthermore, dependency (need for care) was found to be inversely associated with educational level and be a leading contributor to disability among older people with dementia in the 10/66 DRG population‐based surveys conducted in both urban and rural catchment areas in LatAm. These findings have significant implications for the economy and the burden of caregivers.^15^

Taken together, such statistics show that dementia will continue to pose an increasingly significant challenge in LatAm, given the rapid aging of the population and its growing socioeconomic impact. More research is urgently needed to improve the accuracy of the disease estimates in the region. The harmonization of the methodologies used in prevalence, incidence, and mortality studies in LatAm is crucial. Population‐based longitudinal studies with longer follow‐ups are essential to gather evidence of dementia trends in the region.

**Research in context**

1. **Systematic Review**: While Latin America (LatAm) is facing an increasing burden of dementia due to the rapid aging of the population, it remains underrepresented in dementia research, diagnostics, and care. The authors of this article highlight advances in dementia research, diagnosis, and care within LatAm, as showcased during the 2023 Alzheimer's Association International Conference satellite symposium in Mexico City, Mexico.
2. **Interpretation**: Research presented at the symposium highlighted the increasing momentum of dementia research and practices in LatAm. Significant contributions from large initiatives in the region have been instrumental in promoting scientific exchange and fostering national and international collaborations.
3. **Future Directions**: Collaboration and harmonization efforts in LatAm are essential to shape public health policies and foster scientific collaboration. Ongoing efforts in the region have emphasized the importance of intercountry support and the immense potential of international collaborations. A multifaceted approach is imperative in mitigating health disparities in dementia.

## 3 GLOBAL AND NATIONAL DEMENTIA PLAN: EXPERIENCES AND CHALLENGES

### 3.1 Dementia global plan

The World Health Organization (WHO) recognizes dementia as a public health priority and has developed the *Global action plan on the public health response to dementia 2017–2025*.^16^ This comprehensive plan includes seven action areas, providing a guide for policymakers and international and national partners to address dementia globally and regionally.^16^ Despite national and international efforts to meet the targets outlined in the dementia action plan, the WHO's *Global status report on the public health response to dementia in 2021* revealed insufficient progress toward reaching the 2025 target.^17^ Only 50 WHO member states currently have stand‐alone or integrated dementia plans, 43 of which are part of the Global Dementia Observatory (GDO). These figures are significantly less than the 2025 global target, which aims for 75% of the 194 WHO member states to have a dementia plan in place. Furthermore, while two thirds of countries conduct awareness‐raising campaigns or dementia‐friendly initiatives, the majority are concentrated in HICs.^17^ To support the implementation of the dementia global action plan, the WHO has developed valuable resources for each of the action plans (www.globaldementia.org). However, urgent actions are required at the national level to address this pressing issue. This includes implementing policy responses, awareness, risk reduction campaigns, equitable access to diagnosis, treatment, and care services, improving health information systems for dementia, investment in research and innovation strategies, and ensuring equitable inclusion of people with dementia in research and healthcare settings.

### 3.2 Dementia national plans in Latin America

At the 2023 AAIC satellite symposium, Alzheimer's Disease International (ADI, https://www.alzint.org/) noted the importance of integrating civil society organizations in developing and implementing NDPs. ADI's #WhatsYourPlan campaign has gained momentum this year, with 52 country associations and stakeholders participating, 154 official letters, 29 meetings with ministries of health, and 20 commitments to develop NDPs. The ADI experience in past years demonstrated that among the seven action areas in the WHO's dementia global plan, support for dementia carers is particularly important. Supporting dementia carers goes beyond the care they provide for dementia patients but also contributes to capacity building by fostering strong advocates of the NDPs within their community.

In LatAm, seven countries, Chile, Costa Rica, Cuba, Mexico, Puerto Rico, the Dominican Republic, and Uruguay, have already established NDPs, and a plan is under development in Peru, Honduras, and Panama.^18^ In Brazil, the national plan is under Congressional observation, to be approved. However, most countries do not have adequate funding to implement their dementia plans. Mexico was among the first Latin American countries to establish a NDP, but it is facing multiple challenges in the implementation of this plan. Barriers to implementing NDPs in LatAm range from lack of financial resources to social stigma and misconceptions, physician burnout, political challenges, lack of educational resources, and lack of continuity of public policy over time. The National Institute of Geriatrics (NIG) in Mexico has made significant efforts to revise the NDP in Mexico and strengthen the response of the Mexican health system. The 2021 Workshop on Revising the National Plan in Mexico has been particularly important. In this workshop, policymakers and leaders gathered to discuss obstacles and challenges in implementing the NDP in Mexico and identify potential solutions. Accordingly, an updated NDP has been developed to be presented to the policymakers in the health sector in Mexico.

Other topics relating to the NDP in Mexico discussed at the 2023 AAIC satellite symposium included the efforts of the Mexican Federation of Alzheimer's (FEDMA) to combat stigma around dementia (https://www.fedma.mx/). FEDMA, founded in 1988, brings together the different Alzheimer's associations in the Mexican Republic and is a member of ADI and Alzheimer Iberoamerica. An example of FEDMA efforts in Mexico is the Dementia Friends Mexico initiative that aims to change society's perceptions of people with dementia, as those living with dementia experience social exclusion, loneliness, abuse, and neglect. Through Dementia Friends Mexico, it was reported that since 2019, 8500 people have committed to changing their perceptions of people living with dementia. Finally, the dementia caregiver experience was highlighted by the FEDMA chair, who personally cared for her mother, who was diagnosed with dementia in the 1990s. Challenges in caring for her mother were emphasized, including a prolonged diagnostic process and insufficient support from society.

## 4 GENETICS

Characterizing the genetic landscape of ADRD provides a unique opportunity to gain a deeper understanding of the underlying pathophysiology of diseases and develop treatment strategies. While significant progress has been made in unraveling the genetic profiles associated with ADRD,^19^ there remains an inadequate representation of ethnically diverse populations, as most studies have been based on European populations.^20^ Analysis of genome‐wide association studies (GWAS) across human diseases conducted up until 2019 showed that 79% of the GWAS had been conducted in samples of non‐Hispanic European descent, while less than 3% were among admixed Latin American individuals.^20^, ^21^ This disparity hampers the translation of genetic research findings into clinical practice and drives many current efforts to increase diversity in AD genetic studies.^20^ In the 2023 AAIC satellite symposium, a session was dedicated to the genetics of ADRD in LatAm, where researchers discussed topics related to autosomal‐dominant dementia, founder effects, and the impact of ancestry on AD genetics, summarized below.

### 4.1 Autosomal‐dominant Alzheimer's disease: founder effects in Jalisco state and Colombia

Less than 1% of AD cases worldwide have an autosomal‐dominant inheritance pattern and are caused by pathogenic variants in presenilin 1 (*PSEN1)*, presenilin 2 (*PSEN2*), or amyloid precursor protein (*APP*) genes. Of these, *PSEN1* is the most common gene associated with genetic AD, with disease‐causing variants found in approximately 65% of early‐onset familial AD cases, and more than 200 distinct variants have been described in this gene.^22^, ^23^ There are unique characteristics of dominantly inherited Alzheimer's disease (DIAD) families in LatAm, including the presence of common ancestors and suggestive evidence of a high grade of admixture, including African, Western European, Asia, and Native American ancestry.^24^ In LatAm, four major so‐called founder effects – increased prevalence of a specific genetic variant when a new population is established by a small number of individuals – in *PSEN1* have been described, including E280A in Colombia,^25^ G206A in Puerto Rico,^26^ A431E in Jalisco, Mexico,^19^ and M146L in the northeastern region of Argentina.^24^ Each of these variants represents large extended families within LatAm. Below we describe the general characteristics of the most common variants in the region.

The *PSEN1* A431E (rs63750083) was first described by Yescas et al., who reported on nine affected families with early‐onset AD in Jalisco State, Mexico.^27^ Subsequent work identified 15 additional families in Guadalajara (*n* = 2), Chicago (*n* = 1), and Southern California (*n* = 12), nine of which could trace the illness to ancestors from Jalisco.^28^ Haplotype analyses showed that all examined carriers had the same dinucleotide repeat alleles flanking the *PSEN1* gene, suggesting that this variant arose from a common founder.^28^ Currently, more than 100 families with this variant have been identified, and as many as 1260 persons were estimated to be at risk. Systematic phenotyping of the carriers showed that the mean age of onset is around 42 years (±3.9 years), with a high frequency of spastic paraparesis occurring during the first 2 years of the disease.^28^, ^29^ Diffusion tensor imaging (DTI) analyses of *PSEN1* A431E variant carriers with spastic paraparesis showed widespread white matter abnormalities that were not confined to the corticospinal tract, suggesting an effect of the *PSEN1* A431E variant on white matter integrity in the brain.^30^ In the 2023 symposium, it was further highlighted that future work was under way in Mexico, the United States, and in Belgium to characterize the pathologic hallmarks of *PSEN1* A431E variant and develop potential treatments in clinical, imaging, neuropathologic, and animal studies.

The *PSEN1* E280A (rs63750231) variant was first described in 1987 in a Colombian family with early‐onset AD. Subsequent work performed in recent decades identified additional families affected by this variant who primarily live in Antioquia, Colombia. Haplotype analyses of the carriers suggested a founder effect that could be traced back to a founder in the 18th century.^31^ This cohort forms the largest family in the world with autosomal‐dominant AD, consisting of 25 multigenerational families with approximately 6000 family members.^32^ The disease is characterized by several progressive clinical stages, including asymptomatic pre‐mild cognitive impairment (MCI), symptomatic pre‐MCI, MCI, and dementia, with a median age of onset of 35, 38, 44, and 49 years, respectively.^25^, ^33^ However, analyses of more than 300 *PSEN1* E280A variant carriers have shown that the age of onset varies.^34^ While age at onset for most carriers appears to be a polygenic factor, for some distinct outliers, some variants are protective. For example, the apolipoprotein E (*APOE*) ε3ch/ε3ch R154S Christchurch (R136S in the mature protein) variant was observed in a *PSEN1* E280A carrier who did not develop MCI until her seventies.^35^ More recently, the second case of resilience to autosomal‐dominant AD was described in a male carrying a rare RELN variant.^36^ Three more candidate protective variants are currently being studied in this population.

### 4.2 Autosomal‐dominant dementia in DIAN‐LatAm and ReDLat projects

In LatAm, the DIAN‐LatAm study is leading an effort to identify families affected by DIAD.^24^ DIAN‐LatAm is part of the global DIAN observational study (https://dian.wustl.edu/)^37^, ^38^ and is aimed at establishing a Latin American multicenter registry of DIAD families by collecting comprehensive clinical, psychometric, neuroimaging, and biomarker data. Furthermore, the DIAN‐LatAm network aims to create a cohort‐ready population to implement the DIAN‐Trail Unit (DIAN‐TU) platform in the region.^24^, ^39^ The DIAN‐TU platform was launched in 2012 to implement therapeutic trials for DIAD individuals and help in the identification of disease‐modifying agents that can also be translated to sporadic AD.^40^ To date, the DIAN‐LatAm centers include those in Puerto Rico, Mexico, Colombia, Brazil, and Argentina, and new centers are being launched, such as one in Chile. The clinical trial‐ready centers in LatAm within the DIAN‐TU platform include Brazil, while Mexico and Colombia are pending national regulatory approval to initiate trial enrollment in the DIAN‐TU NextGen trial and primary prevention trial. More information about the DIAN‐TU platform within LatAm is available in *Section* *9*.

DIAN‐LatAm has made significant progress in identifying the distribution of at‐risk families, providing resources for genetic testing, and raising awareness about the disease. Notably, 24 DIAD pathogenic variants within LatAm have been reported, with the highest frequencies in Colombia, Puerto Rico, Argentina, and Mexico, which are mostly attributed to founder effects.^24^ Furthermore, five novel variants in the *PSEN1* gene from Brazilian and Mexican families have been identified, four of which were described as likely pathogenic variants.^39^ Collectively, these genetic studies suggest that the frequency of DIAD in LatAm is high, with unique characteristics including common ancestors, large extended families usually related to founder effects, and evidence of a high grade of admixture and ancestry background.^24^

At the 2023 AAIC satellite symposium, the experience of the ReDLat project in genetic screening of autosomal dominant‐like frontotemporal dementia (FTD) was also highlighted. The use of mobile memory clinics in Colombia has led to the identification of large families affected by autosomal dominant‐like FTD who are living in isolated, difficult‐to‐reach areas. The efforts of this mobile memory clinic have created the opportunity to construct large family histories through direct contact with patients and their families. The consortium has identified multiple families with known variants, including *PGN*, *TREM2*, *TARBP*, *MAPT*, *PSEN1*, and *PSEN2*, among others. More details about the ReDLat project are available in *Section* *8*.

### 4.3 Role of ancestry

The genetic diversity of Latin American individuals often results from the process of *admixture*, which is the exchange of genes between populations that had been previously isolated. The makeup of today's populations, particularly in the Americas, represents admixed individuals of European, African, Amerindian, and Asian ancestry, among others. In countries like Colombia, the demographic history of admixture, followed by bottlenecks and founder effects played a significant role in the genetic risk of ADRD. Analyses of genomes from a large sample of Colombian individuals from The Admixture and Neurodegeneration Genomic Landscape (TANGL) study revealed several rare variants associated with ADRD that resulted from founder effects.^41^ These founder effects were present not only for the *PSEN1* variants but also in other genes, such as microtubule‐associated protein tau (*MAPT*). Despite the genetic and environmental similarities among members of families that carry a specific variant, the phenotypic variability in the disease presentation is significant, generating opportunities to better understand risk and resilience factors. For example, the researchers reported how genes traditionally associated with specific phenotypes (eg, *SOD1* and amyotrophic lateral sclerosis) were presenting differently when the variant occurred in other ancestral haplotypes (*SOD1* variant manifesting as FTD when present in Amerindian haplotypes). Therefore, ancestry may significantly impact ADRD phenotypic expression, reinforcing the importance of inclusiveness in genetic studies.^41^

Global and local ancestry can also affect AD risk associated with a particular gene. For example, while  *APOE* ε4 has been shown to substantially increase the risk for AD in Caucasians,^42^ studies across populations with diverse ancestral backgrounds have shown various effect sizes.^43^, ^44^, ^45^ In 2018, Rajabli and colleagues demonstrated that the reason for this difference was due to the surrounding inherited local genomic ancestry.^46^ Specifically, it was shown that among Puerto Rican and African American individuals, only the local ancestral background of the *APOE* alleles influenced AD risk such that the *APOE* ε4 allele on an African ancestral background conferred a lower risk of AD compared to those with a European ancestral background.^46^ Similarly, recent evidence suggests that the effect of the *APOE* ε4 allele on AD is attenuated among Caribbean populations with higher African ancestry proportions.^47^, ^48^, ^49^ A recent study performed in the Peruvian population, known to have up to 80% Amerindian ancestry, suggests that the AD risk conferred by the *APOE* ε4 allele is higher than observed in non‐Hispanic white populations, suggesting a role of the local *APOE* Amerindian ancestry.^50^ A large, one‐of‐a‐kind, clinicopathological study with a Brazilian population showed that people of African ancestry had a lower likelihood of accumulating amyloid plaques than Caucasians and that the *APOE* ε4 allele of African origin does not add an increased risk for AD compared to the *APOE* ε3 allele, as occurs with *APOE* of European origin.^51^ Attenuation of *APOE* ε4 risk in African ancestry was also demonstrated in a clinical cohort from the Caribbean.^51^, ^52^ *Post mortem* analyses of the frontal cortex of AD patients also showed differences in *APOE* gene expression based on diverse ancestral backgrounds.^53^ Ongoing work involves studying the role of ancestry on AD risk from different genes in Peru and Puerto Rico.

Taken together, genetic risk factors for AD can be different depending on ancestral background, and ancestral background can impact phenotypic disease expression. Investigating the relationship between genetic ancestry, social determinants of health, and disease may inform precision medicine initiatives, risk assessment, and the development of ancestry‐specific therapeutics and prevention strategies for ADRD.

## 5 BIOMARKERS

Over the past decade, several biomarkers for AD have been developed that provide valuable insight into the underlying disease pathology. Some of them have been incorporated into revised or updated diagnostic criteria for AD.^54^, ^55^, ^56^ Since these groundbreaking developments in the field, collaborative efforts in LatAm starting in 2011 with the Alzheimer's Disease Neuroimaging Initiative (ADNI) have been instrumental in addressing the challenges of implementing AD biomarkers in the region. A recent survey in LatAm showed a lack of funding as the most important barrier to implementing biomarkers, followed by insufficient infrastructure and training. Despite these challenges, biomarker research in LatAm has witnessed significant advancements, including through the Caribbean Consortium on Dementia (LAC‐CD) and ReDLat initiatives.^57^ At the 2023 AAIC satellite symposium, a session was dedicated to biomarkers, discussing advancements and challenges in biomarker research within LatAm, including neuroimaging, blood‐based, and speech and language biomarkers.

### 5.1 Neuroimaging biomarkers

Neuroimaging is increasingly being utilized as a biomarker to assess dementia in LatAm. A recent online survey in LatAm that was answered by 48 participants from 10 countries showed that neuroimaging is the most widely used biomarker, with magnetic resonance imaging (MRI) being the most commonly used modality, followed by fluorodeoxyglucose (FDG)‐positron emission tomography (PET), DTI, amyloid PET, functional MRI (fMRI), and tau PET. However, widespread implementation of neuroimaging in the region is met with challenges, notably a need for more funding resources. Moreover, degree of experience in using neuroimaging represents another challenge that varies greatly among countries.^57^

New regional initiatives in LatAm are allowing the standardization and harmonization of neuroimaging protocols across different countries while generating high‐quality information. A notable example is the LatAm‐FINGERS study, where significant effort has been expended on developing standardized protocols for use in different countries. Novel MRI quantification techniques, such as quantitative susceptibility mapping, are also being developed that may allow early detection of neurodegenerative diseases.^58^ Other approaches have developed specific methodologies to perform regional comparisons (ie, North vs South) that are robust to multiple levels of heterogeneity.^59^

The Argentina–Alzheimer's Disease Neuroimaging Initiative (arg‐ADNI), established in 2011, was the first ADNI center in LatAm.^60^ Since then, this collaboration has resulted in better characterization of ADRD pathology in LatAm, particularly with the implementation of neuroimaging techniques such as PET and MRI and cerebrospinal fluid (CSF) biomarkers. Currently, various brain PET imaging techniques are being utilized in Argentina, such as amyloid PET (^11^‐C‐PIB and Florbetapir), metabolic PET (^18^FDG), and tau PET (AV1451 and MK6240). Several publications have emerged from this group, highlighting the importance of defining dementias on a biological basis using biomarkers.^61^, ^62^, ^63^, ^64^ For example, in a study of 60 participants from the arg‐ADNI cohort, it was found that 14% of controls, 30% of early MCIs, 53% of late MCIs, and 83% of those with dementia of AD type were amyloid positive using PET imaging (^11^‐C‐PIB). Application of the ATN framework revealed that the conversion rate from MCI to dementia after 5 years of follow‐up was 85% and 50% for A+T+N+ and A−T−N+ patients, respectively.^62^ Furthermore, in the first report from Brazil in a sample from Sao Paulo, 21% of controls, 36% of amnestic MCI, and 74% of AD patients had positive PIB–PET scans.^65^ Despite these advances, AD diagnosis in LatAm remains primarily based on clinical information, while neuroimaging tools such as PET are limited to a few centers.^64^ This reinforces the importance of cross‐regional collaboration in the region to reshape the dementia diagnostic landscape in LatAm.

### 5.2 Blood‐based biomarkers

Blood‐based biomarkers (BBMs) have shown significant promise in revolutionizing the clinical work‐up of dementia patients and improving the design of clinical trials. Compared to well‐established CSF and PET methods, BBMs offer a less invasive, more feasible, and cost‐effective option for application in clinical practice. Additionally, BBMs significantly reduce screening costs and time in clinical trials and may be used as surrogate endpoints to evaluate the effectiveness of therapies.^66^ In recent years, several BBMs have been identified, with the most promising ones being plasma phosphorylated tau (p‐tau), amyloid beta (Aβ) 42/Aβ40, neurofilament light (NfL), and plasma glial fibrillary acidic protein (GFAP).^67^, ^68^, ^69^, ^70^, ^71^, ^72^ For example, plasma p‐tau, especially p‐tau181, is a reliable biomarker for supporting AD diagnosis as it correlates well with amyloid and tau pathology and can differentiate AD from other neurodegenerative disorders.^68^

In LatAm, a recent survey showed that peripheral fluid biomarkers were among the least common dementia biomarkers used in the region.^57^ A systematic review of biomarkers of FTD in LatAm found only a few studies focused on peripheral fluids with a lack of focus on NfL, transactive response DNA binding protein of 43 kDa (TDP‐43), p‐tau, or inflammatory markers.^73^ Therefore, utilization of BBMs remains limited in LatAm despite their high potential to improve diagnostic and clinical work‐up. Efforts from different regional initiatives are under way to pave the way to BBM use in clinical and research settings, including LAC‐CD, ReDLat, and The Latin American Institute of Brain Health (BrainLat). There is an urgent need to promote and facilitate the use of BBMs in LatAm.

### 5.3 Speech and language biomarkers

While discussions of linguistic deficits in dementia are often restricted to primary progressive aphasia (PPA), verbal dysfunction is common across neurodegenerative disorders. For example, the prevalence of speech and language deficits is 36% to 90% in early‐stage AD and 60% to 90% in early‐stage Parkinson's disease (PD). In early‐stage behavioral‐type FTD (bvFTD), this figure ranges from 5% to 55% depending on the language domain affected.^74^ Therefore, speech and language profiles are promising markers of neurodegenerative disorders that can provide novel complementary information to the standard biomarkers.

Researchers from LatAm have developed novel automated approaches using artificial intelligence and natural language processing to automatically capture speech and language alterations in neurodegenerative disorders from patients’ audio recordings. In a study of Chilean participants, machine learning analyses of natural speech data showed that semantic granularity and semantic variability discriminated between AD individuals and controls with an area under the curve (AUC) of 80% while yielding near‐chance classifications between PD patients and controls.^75^ Moreover, automated analysis of word properties in verbal fluency tasks can identify Latino AD patients with an AUC of 89%, predict their neuroanatomical and neurofunctional abnormalities, and differentiate them from bvFTD patients.^76^ Other studies with automated tools have revealed novel speech and language markers of cognitive decline in Latin American PD cohorts.^77^, ^78^ Therefore, such automatic speech and language markers may contribute to the identification, differentiation, and characterization of diverse neurodegenerative disorders.^79^ In the 2023 AAIC Satellite Symposium, various initiatives from LatAm have been highlighted that aim to foster international collaborations to study speech and language alterations in neurological disorders, such as the International Network for Cross–Linguistic Research on Brain Health (Include network) and NIH‐funded projects in the region. Moreover, a novel web‐based app, called TELL, has been developed specifically to capture such markers in Latino populations.^80^ Promisingly, these efforts address the urgent call to increase linguistic diversity and equity in the field.^81^

## 6 BIOBANKS

Biobanks are invaluable resources in the study of neurodegenerative disorders by providing insights into population‐specific risk and protective factors and helping determine the prevalence of dementia subtypes. Such information is critical to guide and shape public health policy efforts. Furthermore, biobanks provide excellent opportunities to advance our understanding of the pathological underpinnings of dementia. However, most biobanks are in HICs despite the fact that most people living with dementia reside in LMICs.^82^ Data from emerging biobanks in LatAm has shown the importance of biobanking in LMICs as they can provide valuable insights into disease mechanistic pathways in the context of diverse ethnoracial backgrounds.

A notable example of brain biobanks in LatAm is the Biobank of Aging Studies (BAS) in Sao Paulo, Brazil. Since its inception in 2003, the BAS has aimed at maximizing available resources in the region and providing high‐quality material for multidisciplinary researchers to advance knowledge of age‐related disorders of the brain. The BAS follows international standards and principles, especially standardized protocols from the Netherlands Brain Bank.^83^ Leveraging BAS resources, important discoveries into the prevalence and pathology of ADRD in the region have been made. For example, while over 60% of cases in the BAS have a Clinical Dementia Rating (CDR) score of 0, neuropathological examination showed that the prevalence of vascular dementia (VaD) is substantially high, with small‐vessel disease present in 39% of all dementias and 17% of those without cognitive impairment.^84^, ^85^ Other studies from the BAS showed that genetically determined African ancestry individuals tended to accumulate significantly fewer neuritic plaques, even among individuals who self‐declared as White.^86^ Another larger follow‐up study showed that a higher African ancestry proportion in AD patients was associated with lower plaque accumulation in the brain. Interestingly, however, after normalizing the number of plaques across individuals, African ancestry was associated with worse cognitive function. Further analyses of genotypes revealed that the relationship between higher African ancestry proportion and worse cognition disappeared in those with the *APOE* ε4 genotype.^51^ These findings underscore the interaction between ancestral background and *APOE* genotype in AD risk in diverse populations.

The Argentina brain bank is another example that was highlighted in the 2023 AAIC satellite symposium. The survey of prion disease cases served as the foundation of establishing this biobank, with a report of the first 10 neuropathologically confirmed Creutzfeldt–Jakob disease cases published in the 1980s.^87^ Other notable findings from the Argentina biobank include the first reports of an Argentine kindred affected by Gerstmann–Sträussler–Scheinker syndrome,^88^ insomnia associated with thalamic involvement in E200K Creutzfeldt–Jakob disease,^89^ and a case of MM1+2C sporadic Creutzfeldt–Jakob disease presenting as rapidly progressive non‐fluent aphasia.^90^ Resources from this biobank were further instrumental in developing the final diagnosis of neuropathologically complex disorders.^91^ Currently, the biobank hosts tissue from 149 cases of prion disease and 48 cases of other types of dementia.

Other notable biobanks in LatAm that were highlighted at the symposium were the Colombian brain bank known as the Neurobanco, the National Dementia Biobank (BND) in Mexico, and the DNA Bank Neurogenetics in Peru. Neurobanco is part of the Antioquia Neuroscience Group (GNA) and is located at the University Research Headquarters (SIU) of the University of Antioquia (Medellín—Colombia). It was established in 1995, is focused on the study of neurodegenerative diseases, and has the largest repository of brains from a family group with AD (more than 140 brains from those affected with the E280A mutation in *PSEN1*). Also, it has multiple brains with a wide repertoire of neurodegenerative diseases. Along with brains, Neurobanco has stored more than 20,000 samples of DNA, serum, plasma, CSF, and other samples. For several years, it has focused on the quality of its processes, leading to extensive clinical information on each of its cases, photographic records, a digital neuropathology system, protocols for cultures of nerve cells from donors, and a donation program that favors social interaction with donors’ families and short *post mortem* times. Neurobanco, among its many studies, has helped with the pathophysiological characterization of neurodegenerative diseases, the identification of protective factors for AD, and the molecular analysis of factors related to neurodegenerative diseases.^36^, ^92^, ^93^, ^94^, ^95^ Neurobanco, together with the other brain banks in LatAm, created the “Latin American and Caribbean Brain Bank Network” and supports the creation of other brain banks in LatAm, such as the brain bank of Peru and Chile. Dr. Raúl Mena López founded the BND 30 years ago, making it the oldest biobank in LatAm. The BND is a diagnostic and research unit in the Polytechnic University of Pachuca that has as its aim supporting the confirmatory diagnosis of neurodegenerative diseases. The BND has focused on research on biomarkers and non‐invasive methods for the early diagnosis of AD.^96^ The BND highlighted collaboration with several entities, such as the Latin American and Caribbean Brain Bank Network, the Brain Bank of the Dominican Republic, the Brain Bank of Peru, DIAN, and several academic institutions. DNA Bank Neurogenetics, founded in 2020, became the first DNA biobank of the public healthcare system in Peru. It supports various collaborative research projects, such as the Latin American Research Consortium on the Genetics of Parkinson's Disease (LARGE PD) and the Peruvian Alzheimer's Disease Initiative (PeADI). However, developing local capacities, long‐term sustainability, and recruitment of participants have been the most relevant challenges faced by this Peruvian DNA bank.^97^

## 7 MODIFIABLE RISK FACTORS FOR DEMENTIA AND VULNERABLE POPULATIONS

While 12 potentially modifiable risk factors across the life course have been shown to account for 40% of dementia cases worldwide,^98^ the population attributable fraction (PAF) differs between LMICs and HICs.^99^ The 10/66 DRG data found that in LatAm, 56% of dementias are attributed to modifiable risk factors, whereas the PAF in the United States is around 30%.^2^, ^99^ In Brazil, 48% of all‐cause dementias are attributed to the 12 modifiable risk factors, with low education, midlife hearing loss, hypertension, and obesity being among the most important ones.^100^ These figures suggest that targeting modifiable risk factors for dementia may give more room for dementia prevention in LMICs, including in LatAm, than in HICs.^2^ Furthermore, health communication campaigns should consider including dementia as a possible consequence of chronic diseases. Recent studies, utilizing methodological innovations and larger datasets, have shown that social and health disparities are the primary drivers of many significant risk factors in the entire region, underscoring the need for more sensitive and tailored models.^101^, ^102^

To address the role of dementia risk factors in LatAm, the 2023 AAIC satellite symposium devoted a session to dementia risk factors in vulnerable populations. Researchers from ReDLat highlighted their ongoing work on assessing the association between social determinants of health and brain health, researchers from Peru highlighted their study on assessing the impact of socioeconomic status, lifestyle factors, and healthcare providers on cognitive function in indigenous people in Peru, and researchers from Argentina highlighted their work on gender‐specific modifiable risk factors for dementia. In addition, the roles of education and cardiovascular risk factors were highlighted and are summarized below.

### 7.1 Education and literacy

Mounting evidence from HICs suggests that dementia is more common among illiterate populations, and higher education is associated with lower dementia risk. In LatAm, the prevalence of dementia is two times higher in illiterate than literate individuals.^11^, ^103^ Even more striking is the incidence of dementia, which has been shown to be five times higher in illiterate people compared to literate populations in Brazil.^14^ In the BAS, education, but not occupation, was associated with better cognitive function as measured by the CDR sum of boxes.^100^ A cross‐sectional door‐to‐door study in Puente Piedra, one of the most socially and economically vulnerable districts of Lima, the capital of Peru, reports that among 247 participants with a median age of 46 years, one‐fourth had not completed secondary school and more than 50% had completed only secondary school. In this cohort, there was a high frequency of possible neurocognitive disorders (NCDs), with younger adults showing levels of NCD higher than expected.^104^ The mechanism underlying the relationship between illiteracy and dementia risk has been explored by researchers from LatAm using neuroimaging tools. One study with 31 participants showed that literate older adults had better white matter integrity, meaning stronger brain connections, than their illiterate counterparts.^105^ Later, this finding was replicated in a large cohort of more than 600 US middle‐aged individuals in which literacy levels were also associated with better white matter integrity, especially in temporal and parietal regions.^106^ Furthermore, because the hippocampus is one of the most plastic regions of the brain and plays an important role in episodic memory, it has become the target of investigation on the mechanisms of cognitive reserve in low literacy populations. It has been shown that the relationship between hippocampal volume and episodic memory seems to be moderated by educational level.^107^, ^108^

### 7.2 Cardiovascular risk factors

VaD is the second most common cause of dementia after AD, accounting for 15% of dementia cases in HICs. However, emerging data suggest that the burden of VaD in LMICs is higher than in HICs.^109^ In LatAm, results from the BAS revealed a prevalence of VaD of 35%, and this figure could reach 49% if the diagnostic criteria included neuropathologic features of small‐vessel disease.^84^ In line with this, the prevalence of cardiovascular risk factors in LatAm remains high. The Brazilian Longitudinal Study of Adult Health showed that 53% of participants had poor cardiovascular health as measured by the American Heart Association's Life Simple 7 criteria.^110^ Importantly, this study showed that poor cardiovascular health was associated with worse performance on several domains of cognitive function such as executive function, language, and memory.^111^ In Chile, it has been reported that the proportion of dementia associated with modifiable risk factors is 45.8%, with high blood pressure, obesity, and hearing loss being the most important modifiable risk factors.^112^ These findings underscore the importance of effective management of cardiovascular risk factors in LatAm as these factors play important roles in mitigating the rising burden of dementia in the region.

## 8 HARMONIZING LARGE DATA AND DEVELOPING COLLABORATIVE RESEARCH

Establishing effective guidelines for data harmonization, data collection, and data sharing is essential to adopting methodologies and interventions according to best clinical practice. These guidelines are instrumental in creating robust clinical cohorts and research studies that advance our knowledge of diseases, especially neglected or rare diseases.^113^ Furthermore, data harmonization and sharing guidelines can help guide public health policies and foster scientific collaboration. However, harmonizing large amounts of data and developing collaborative research in LatAm face considerable challenges. A recent study in the region showed that factors such as cultural, linguistic, socioeconomic, and educational diversities, as well as limited access and coverage of biomarkers and dementia awareness and stigma, remained significant barriers to collaborative dementia research in LatAm.^5^ Despite these difficulties, ongoing regional initiatives, such as the Brazilian Registro Brasileiro de Doenças Neurológicas (REDONE) databank of neurological diseases,^114^ since 2003 the 10/66 DRG protocols, the ReDLat project (https://red‐lat.com/), DIAN‐LatAm, and the LatAm‐FINGERS study^115^ have shown the potential of collaborative research and large‐scale data harmonization procedures in LatAm. For example, ReDLat has developed post‐recording harmonization procedures using different computational methods for clinical and cognitive data,^116^ neuroimaging,^59^, ^117^ epidemiological data,^101^, ^118^ and assessment of various measures of socioeconomic disparities.^119^ Furthermore, collaboration and harmonization efforts in LatAm need to pursue the development of accessible and scalable cognitive and functional screening tools to help advance both clinical and research efforts. Recent regional developments in the region are making strides in addressing this gap.^120^, ^121^, ^122^

### 8.1 Data harmonization and collaborative research in Latin America: 10/66 DRG, RedDLat, LatAm‐FINGERS, and DIAN LatAm initiatives

In 2000, the 10/66 DRG was established with the aim of addressing the worldwide imbalance in epidemiological research on dementia in LMICs. The name “10/66” symbolizes the situation at that time, where more than two‐thirds (66%) of the population with dementia were living in LMICs, while only 10% of the epidemiologic research originated from these regions. The 10/66 DRG program utilizes population studies, with standardized protocols and training across all centers. It is primarily focused on the evaluation of mental health among the elderly population, with an emphasis on dementia. The 10/66 DRG protocols have come in three waves: baseline evaluation or prevalence phase (2003 to 2009), first follow‐up approximately 3 to 5 years later, and a second follow‐up approximately 10 years later, and ongoing to present. This initiative has produced more than 200 papers, with more than half involving data from LatAm.

ReDLat^121^ is a 5‐year project aimed at expanding dementia research in LatAm and the Caribbean region by utilizing socioeconomic, genomic, neuroimaging, and behavioral measures in diverse populations. This project has created an unprecedented opportunity to foster multidisciplinary research to promote harmonizing global strategies to treat and prevent dementia in underserved populations. Since its establishment, harmonized and cross‐regional approaches across 13 sites from seven countries on the American continent have led to several achievements. Notably, ReDLat has produced over 150 publications in the top scientific journals and provided 12 seed grants fostering 22 projects through the BrainLat Institute. Examples of ongoing ReDLat research projects are the genomic data showing the level of genetic admixture in LatAm,^123^ machine learning approaches capable of minimizing sources of heterogeneity in cognitive assessment tools,^116^ development of fully automated approaches to analyzing whole‐brain neuroimaging data,^59^ assessing the impact of social determinants of health on dementia,^124^ developing novel theoretical models to understand the biological underpinning of dementia,^125^, ^126^ and development of educational resources for brain health and dementia in the region.

LatAm‐FINGERS is an ongoing multidomain lifestyle intervention trial for dementia prevention in Latin American countries that are often underrepresented in dementia clinical trials.^115^, ^127^ Extensive harmonization processes and protocols have been implemented both externally with the original FINGERS (Finnish Geriatric Intervention Study to Prevent Cognitive Impairment and Disability)^128^ and US Study to Protect Brain Health Through Lifestyle Intervention to Reduce Risk (US POINTER, https://alz.org/us‐pointer/home.asp) trials and internally across Latin American countries. The harmonization processes extended to interventions by adopting the FINGERS trial concept while considering cultural differences and affordability factors. For example, a new LatAm MIND diet was established that includes core components that must be respected (nutrient components and portions) while allowing ingredient flexibility. The outcome measures were harmonized by selecting variables from FINGERS and US POINTER trials while allowing alignment with linguistics differences and ensuring validity for comparisons within LatAm and externally. Various outcome measures are being collected, such as blood and DNA samples, neuroimaging, and cognitive and clinical assessments. The LatAm‐FINGERS data are embargoed for 2 years, after which data will be available to the scientific community through the Global Alzheimer's Association Interactive Network (GAAIN) platform (https://www.gaain.org/). More information on LatAm‐FINGERS is provided in *Section* *9.1*.

## 9 PHARMACOLOGICAL AND NON‐PHARMACOLOGICAL INTERVENTIONS

Individuals living in LMICs are often underrepresented in dementia clinical trials, limiting the relevance of clinical trials conducted in HICs to LMICs, due to factors such as ethnic, socioeconomic, and educational differences, as well as variations in disease profiles and healthcare access. A study of AD clinical trials in 2013 showed that among 715 worldwide AD clinical trials, only 34 were performed in South America.^129^ Similarly, a 2020 study showed that only 6% of dementia clinical trials occurred in LatAm, primarily in Brazil, Argentina, Chile, Mexico, and Colombia.^5^ A recent systematic review of the distribution of ADRD clinical trials showed that less than 3% of trials are conducted in LatAm and Africa.^130^ These figures highlight the inequity in ADRD clinical trials in LMICs, despite the fact that these regions bear the greatest burden of dementia. Between 2013 and 2022, the API Colombia study was carried out in Colombia, one of the most important clinical trials of secondary prevention for AD demonstrating the ability to carry out this type of study in the region. LatAm‐FINGERS is an example of non‐pharmacological interventions in the region, underscoring the need for more studies on non‐pharmacological interventions in the region.

Recent developments in AD‐modifying interventions have opened up a new landscape in AD diagnosis, care, and treatment.^131^ However, the application of such therapies to LatAm is challenged by factors such as delay in MCI and dementia diagnosis, a higher burden of neurovascular pathologies in Latinos,^84^ costs, and a shortage of dementia specialists. For example, a study in Brazil found that it took 1.5 years for patients to receive a dementia diagnosis, mainly due to physicians' inability to reach a conclusion and families attributing symptoms to normal aging.^132^ Furthermore, a systematic review of AD biomarkers found significant discrepancies between developed and developing countries.^133^ These findings highlight the need for targeted and concentrated efforts in LatAm to improve early dementia diagnosis, raise dementia awareness, and establish infrastructure toward developing and implementing dementia disease‐modifying interventions.

In the 2023 AAIC satellite symposium, ongoing pharmacological and non‐pharmacological interventions for dementia in LatAm were highlighted, including LatAm‐FINGERS, DIAN‐TU, and API Colombia, which are summarized below.

### 9.1 LatAm‐FINGERS

The LatAm‐FINGERS study is part of the World Wide FINGERS Network funded by the Alzheimer's Association (https://www.alz.org/wwfingers/overview.asp). LatAm‐FINGERS is a non‐pharmacological, multicenter, randomized clinical trial (RCT) in 12 Latin American countries (Argentina, Bolivia, Brazil, Chile, Colombia, Costa Rica, Dominican Republic, Ecuador, Mexico, Peru, Puerto Rico, and Uruguay) assessing the feasibility and efficacy of a multidomain lifestyle intervention on cognitive function. The aim is to enroll 1200 individuals (100 from each country) aged 60 to 77 years who are at a high risk of cognitive deterioration due to a sedentary lifestyle and suboptimal metabolic‐cardiovascular profile. The two lifestyle interventions include the Systematic Lifestyle Intervention (SLI) and Flexible Lifestyle Intervention (FLI). The multidomain lifestyle intervention, or SLI, consists of adherence to the LatAm‐MIND diet, physical exercise, cognitive training, and control of cardiovascular risk factors, while the FLI includes regular general health advice. Participants will be evaluated every 6 months to collect data on clinical and neuropsychological assessments, blood samples, and brain MRI.^115^ The major challenges experienced by LatAm‐FINGERS have been training team members across 12 countries and finding adequate facilities to perform interventions, and harmonization efforts (as described in *Section* *8.1*). Adapting the dietary intervention and cognitive training to LatAm was particularly challenging. For example, new foods, such as avocado replacing olive oil, were incorporated into the LatAm‐MIND diet only after confirming their nutritional profile equivalence to the MIND diet. Furthermore, customized solutions have been established to ensure participants' adherence to interventions, especially the computerized cognitive training component.^115^ Collectively, the LatAm‐FINGERS study provides a groundbreaking opportunity for collaborative studies in LatAm, generating valuable data from a region often underrepresented in dementia RCTs. These data are poised to significantly impact the development of health policies for dementia prevention in LatAm.

### 9.2 DIAN‐TU

The aims of DIAN‐TU are to execute effective preventive and treatment strategies for DIAD, determine the timing of AD treatment for improved clinical outcomes, identify changes in biomarkers to track therapeutic effectiveness, and test AD hypotheses through treatment trials. The DIAN‐TU platform has been split into two main trial design approaches: the primary prevention trial for treating participants up to 11 years before the estimated age of AD onset and the secondary prevention trial for those within ±10 years of the estimated age of AD onset.^37^, ^38^, ^134^ In 2012, the first DIAN‐TU trial was launched, where two monoclonal antibodies of gantenerumab and solanezumab were tested in asymptomatic and symptomatic patients. Gantenerumab showed a clear reduction in brain amyloid accumulation and improvement in other downstream biomarkers of AD. However, gantenerumab and solanezumab did not meet the primary endpoint of improving cognitive function.^135^ Therefore, alternative treatments are being explored for the primary prevention trial. In the secondary prevention trial (Tau NextGen trial),^40^ two drugs will be tested: Lecanemab^136^ and a new antitau antibody, E2814.^137^ Eligible participants with a known mutation on *PSEN1*, *PSEN2*, and *APP* will be grouped based on CDR scores into symptomatic (cohort 1) or asymptomatic (cohort 2) cohorts. The order of intervention randomization will depend on these cohorts, with two main combinations of Lecanemab+E2814 or Lecanemab+placebo at the end of the first year. The trial endpoints are stage‐specific, with tau PET being the primary outcome for cohort 1 (symptomatic) and CSF tau biomarkers being the primary outcome for cohort 2 (asymptomatic). In LatAm, Argentina has started trial enrollment, Brazil recently received approval to start the DIAN‐TU trial, while Mexico, Colombia, and other countries are under way. A remarkable aspect of DIAN‐TU in LatAm is the collaborative and supportive work across countries, such as Colombian participants undergoing PET in Argentina. The DIAN‐LatAm initiative has supported technology and tracer development at imaging centers (Argentina and Mexico) across the trial network.

### 9.3 API ADAD Colombia

The Alzheimer's Prevention Initiative Autosomal Dominant Alzheimer's Disease Colombia Trial (API ADAD Colombia) was launched in 2013 to evaluate the efficacy, safety, and tolerability of crenezumab (an anti‐Aβ drug) in cognitively unimpaired E280A mutation carriers.^138^ The study was conducted at the University of Antioquia in Medellín, Colombia, with satellite sites in Yarumal, Bogota, and Armenia for dosing and safety assessments. A total of 252 individuals were enrolled, including 169 E280A mutation carriers and 83 noncarriers, and enrollment ended in 2017. The E280A mutation carriers were randomized to active treatment or placebo, and non‐carriers received placebo.^138^ The modifications in the API trial led to the dose of crenezumab increasing more than seven‐fold throughout the study. The primary outcome was the annualized rate of change in API ADAD composite score. Key secondary outcomes were changes in amyloid PET, time to progression to MCI or dementia due to AD, and changes in dementia severity and neurocognitive functioning. The study showed excellent adherence and retention rates over 8 years, with 90% of participants completing the treatment. Results numerically favored crenezumab across primary and secondary outcomes, but they did not reach statistical significance.^139^, ^140^ While the API ADAD Colombia trial results were negative, it led to the discovery of protective genetic variants^35^, ^36^, ^94^ and additional pathogenic *PSEN1* mutations in Colombia,^41^ as described in more detail in *Section* *4* of this manuscript.

## 10 CONCLUSION

The myriad of topics discussed at the 2023 AAIC satellite symposium highlighted the growing research efforts in LatAm, providing valuable insights into dementia biology, genetics, epidemiology, treatment, and care. Large research initiatives, such as 10/66 DRG studies, LAC‐CD, RedLat, DIAN‐LatAm, and LatAm‐FINGERS studies, have played critical roles in promoting regional scientific exchange and fostering national and international collaborations. Such initiatives have provided unique insights into the etiology of AD in LatAm and paved the way toward tailored interventions suited to the needs of LatAm's diverse and culturally rich populations. Furthermore, genetic studies conducted in the region have significantly enriched our understanding of the variation and complexity of dementia risk and resilience factors and the importance of genetic ancestry. These findings hold the promise of global translatability, offering hope for developing effective preventive or treatment strategies for dementia.

## CONFLICT OF INTEREST STATEMENT

S. Mahinrad, C. E. Sexton, and M. C. Carrillo are full‐time employees of the Alzheimer's Association. A. L. Sosa reports receiving in the past 36 months consulting fees from Grupo Toscana; receiving payment or honoraria for lectures, presentations, speakers’ bureaus, manuscript writing, or education events from Aphopharma, Biogen, Carnot, and Lundbeck; and participating in a data safety monitoring board or advisory board of Roche. S. M. D. Brucki reports receiving in the past 36 months consulting fees from Biogen and Roche; payment for lectures at Aché Pharmaceuticals and Novo Nordisk; and support from Novo Nordisk to attend meetings. L Crivelli reports receiving in the past 36 months reimbursement for travel expenses as a member of the Scientific Program Committee to attend AAIC Satellite Mexico 2023, AAIC Amsterdam 2023, and as part of the IMPACT‐AD fellowship from Alzheimer's Association and IMPACT‐AD. F. J. Lopera reports receiving in the past 36 months fees for consulting to Dr. Joseph Arboleda in the Alzheimer's Resistance program, receiving patent Antibody APOE3Ch, and participating on the advisory board of VIEW MIND. D. Acosta reports receiving in the past 36 months support for attending the 2023 AAIC Satellite Symposium in Mexico. J. A. Uribe reports receiving in the past 36 months payment or honoraria from Cajal Neuroscience and Regeneron and support for attending meetings from the University of California Santa Barbara and Rainwater Charitable Foundation. P. H. F. Bertolucci reports receiving in the past 36 months consulting fees from Novo Nordisk, Knight Laboratories, and Support‐Danone; payment from Novo Nordisk for lectures, presentations, speakers’ bureaus, manuscript writing, or educational events; and support from Novo Nordisk for attending meetings. I. L. Calandri reports receiving in the past 36 months support from the Alzheimer's Association to attend AAIC Satellite Mexico 2023. M. C‐Olivas reports receiving in the past 36 months payment or honoraria from MDS for lectures, presentations, speakers’ bureaus, manuscript writing, or educational events; support from National Institutes of Health (NIH) subcontract and ASAP/GP2/MJF for attending meetings; and serving on the MDS‐PAS Executive Committee and as regional mentor for GBHI Fellowship and President of Rare Disorders National Board. N. Custodio reports receiving support to attend the AAIC Satellite Symposium, Mexico 2023. A. Damian reports receiving support to attend the AAIC Satellite Symposium Mexico 2023. L. C. de Souza reports receiving in the past 36 months payment for the development of continuing medical education material and participation as a speaker in symposia sponsored by Biogen and Abbott and payment for serving on the advisory board for Biogen and Lilly. M. García reports receiving financial support from TELL SA and receiving support from Alzheimer's Association to attend AAIC Satellite Mexico 2023. M. M. Gonzales reports having personal stock in AbbVie. L. T. Grinberg reports receiving in the past 36 months consulting fees from Guidepoint Global Inc.; receiving payment or honoraria from Medscape Inc. and Celdare Medical for lectures, presentations, speakers’ bureaus, manuscript writing, or educational events; receiving support for attending meetings and/or travel from the Alzheimer's Association and BrightFocus Foundation; and serving in leadership or fiduciary roles in Global Brain Health Institute. M. Z. I‐M. reports receiving in the past 36 months support for attending meetings and/or travel from the Alzheimer's Association and Global Brain Health Institute. J. M. Leon‐Salas reports receiving in the past 36 months support for attending meetings and/or travel from the Alzheimer's Association. B. L. Miller reports receiving in the past 36 months payments for royalties or licenses from Cambridge University Press, Elsevier, Inc., Guilford Publications, Inc., Johns Hopkins Press, Oxford University Press, and Taylor & Francis Group; consulting fees from Massachusetts General Hospital Alzheimer's Disease Research Center (ADRC) Scientific Advisory Board (SAB), Stanford University ADRC SAB, University of Washington ADRC SAB, and Genworth Medical Advisory Board; receiving payments from the Institute for Lifelong Learning, Global Summit on Neurodegenerative Diseases, Korean Dementia Society, Massachusetts General Hospital, dementia course, National MS Society, Don Paty Lectureship, Ochsner Neuroscience Institute, Providence Saint Joseph Medical Center, Taipei Medical University, Dementia Center, UC Irvine Institute for Memory Impairments and Neurological Disorders (UCI MIND), University of California, Los Angeles (UCLA) Grand Round, University of Texas, and Center for Brain Health for lectures, presentations, speakers’ bureaus, manuscript writing, or educational events; receiving support for attending meetings and/or travel from the Association for Frontotemporal Degeneration (AFTD) Education Symposium, St. Louis, MO, Milken Institute FTD Scientific Retreat, Los Angeles, CA, California Institute of the Arts, Los Angeles, CA, and UCLA; serving on a Data Safety Monitoring Board or Advisory Board of Arizona Alzheimer's Consortium, Association for Frontotemporal Degeneration, The Buck Institute for Research on Aging, Cure ALS, The John Douglas French Alzheimer's Foundation, Fundación Centro de Investigación Enfermedades Neurológicas, Madrid, Spain, Genworth, The Larry L. Hillblom Foundation, Massachusetts General Hospital ADRC, National Institute for Health Research Cambridge Biomedical Research Center and its subunit, the Biomedical Research Unit in Dementia, Stanford University ADRC, University of Southern California P01 Urban Air Pollution and Alzheimer's Disease: Risk, Heterogeneity, and Mechanisms, and University of Washington ADR; having a leadership or fiduciary role in the Bluefield Project to Cure FTD, Global Brain Health Institute, Institute for Neurodegenerative Diseases and Tau Consortium of the Rainwater Charitable Fdtn. L. Naci reports receiving in the past 36 months support from the Global Brain Health Institute for attending meetings and/or travel. M. A. Parra reports receiving in the past 36 months consulting fees from ViewMind. E. D. P. F. Resende reports receiving consulting fees from United Medical Ltda in 2021, payment or honoraria from Proneuro, NovoNordisk, and Roche for lectures, presentations, speakers’ bureaus, manuscript writing, or educational events; receiving support for attending meetings and/or travel from the Alzheimer's Association; serving as the deputy chair of the Scientific Department for Behavioral Neurology and Aging at the Brazilian Academy of Neurology. J. M. Ringman reports receiving in the past 36 months consulting fees from Eisai Pharmaceuticals, payments from PriMed CME/CE organization for lectures, presentations, speakers’ bureaus, manuscript writing, or educational events; support from the Alzheimer's Association to attend the AAIC Satellite Mexico 2023; and doses of flortaucipir for research from Avid Pharmaceuticals. G. Sevlever reports receiving support for attending meetings and/or travel from Fleni, Buenos Aires, Argentina. A. Slachevsky reports receiving support from the Alzheimer's Association and GBHI for attending meetings and/or travel and having a leadership or fiduciary role on the board of Global Brain Health Institute. C. K. Suemoto reports receiving a travel fellowship from the Alzheimer's Association to attend the 2023 AAIC Satellite Symposium and serving on the ISTAART advisory council and in Brazilian Society of Geriatrics and Gerontology. V. Valcour reports receiving payment or honoraria from IAS‐USA for providing CME; and support from the Atlantic Philanthropies for attending meetings and/or travel. M. S. Yassuda reports payment or honoraria from Supera – Brazilian Company and Institute of Neurology – BRAIN Congress in Brazil for lectures, presentations, speakers’ bureaus, manuscript writing, or educational events amd support from Brazilian National Council for Scientific and Technological Development and the Alzheimer´s Association to attend AAIC in Mexico.

The remaining authors have nothing to declare. Author disclosures are available in the supporting information.

The contents of this publication are the sole responsibility of the authors and do not represent the official views of these institutions.

## CONSENT STATEMENT

Consent was not necessary.

## Supporting information

Supporting Information
